# Adaptive homeostasis and the p53 isoform network

**DOI:** 10.15252/embr.202153085

**Published:** 2021-11-15

**Authors:** Sunali Mehta, Hamish Campbell, Catherine J Drummond, Kunyu Li, Kaisha Murray, Tania Slatter, Jean‐Christophe Bourdon, Antony W Braithwaite

**Affiliations:** ^1^ Department of Pathology School of Medicine University of Otago Dunedin New Zealand; ^2^ Maurice Wilkins Centre for Biodiscovery University of Otago Dunedin New Zealand; ^3^ Dundee Cancer Centre Ninewells Hospital and Medical School University of Dundee Dundee UK

**Keywords:** homeostasis, immune response, inflammation, p53 isoforms, pathogen, Autophagy & Cell Death, Immunology, Signal Transduction

## Abstract

All living organisms have developed processes to sense and address environmental changes to maintain a stable internal state (homeostasis). When activated, the p53 tumour suppressor maintains cell and organ integrity and functions in response to homeostasis disruptors (stresses) such as infection, metabolic alterations and cellular damage. Thus, p53 plays a fundamental physiological role in maintaining organismal homeostasis. The *TP53* gene encodes a network of proteins (p53 isoforms) with similar and distinct biochemical functions. The p53 network carries out multiple biological activities enabling cooperation between individual cells required for long‐term survival of multicellular organisms (animals) in response to an ever‐changing environment caused by mutation, infection, metabolic alteration or damage. In this review, we suggest that the p53 network has evolved as an adaptive response to pathogen infections and other environmental selection pressures.

## Graphical abstract

The TP53 gene encodes a network of isoforms with distinct functions. This review provides an overview on the p53 network and suggests that it has evolved as an adaptive response to pathogens and other environmental pressures.

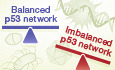

**Glossary**
AKT: Protein kinase B
AMPK: AMP‐activated protein kinase
CCL2: Chemokine (C‐C motif) ligand 2
CHARGE: Coloboma, Heart defects, Atresia choanae (also known as choanal atresia), growth Retardation, Genital abnormalities and Ear abnormalities
CPSF4: Cellular protein cleavage and polyadenylation specificity factor 4
DDX5: DEAD box protein 5
DHX15: DEAH‐Box Helicase 15
DNA: Deoxyribonucleic acid
dNTPs: Deoxyribonucleotide triphosphates
EBV: Epstein–Barr Virus
EGR1/KLF5: Early growth response protein 1/Kruppel‐like factor 5
eIF2α: Eukaryotic translation initiation factor 2‐alpha
ERV: Endogenous RetroViruses
GM‐CSF: Granulocyte‐macrophage colony‐stimulating factor
HASMCs: Human aortic smooth muscle cells
HGF: Hepatocyte growth factor
HPV: Human papilloma viruses
IAV: Influenza A virus
IFITM: Interferon‐induced transmembrane protein
IFNs: Interferons
IL‐10: Interleukin‐10
IL‐6: Interleukin‐6
iPSCs: Induced pluripotent stem cells
JAK/STAT: Janus kinases/ signal transducer and activator of transcription proteins
JNK: c‐Jun N‐terminal kinase
LINE: Long INterspersed Elements
LTR: Long terminal repeat
MAPK: Mitogen‐activated protein kinase
MDM2: Mouse double minute 2 homologue
MEF: Mouse embryonic fibroblasts
mRNA: messenger RNA
NF‐κB: Nuclear factor kappa B
NS1: Non‐structural protein 1
p16INK4A: Cyclin‐dependent kinase 4 inhibitor
p21cip1: Cyclin‐dependent kinase inhibitor 1
PD‐1: Programmed cell death protein 1
PD‐L1: Programmed cell death 1 ligand 1
PI3K: Phosphatidylinositol 3‐kinase
PUMA: p53 upregulated modulator of apoptosis
Rb: Retinoblastoma protein
RCHY1: Ring Finger And CHY Zinc Finger Domain Containing 1
RE: Response element
ROCK: Rho‐associated protein kinase
ROS: Reactive oxygen species
SAPK: Stress‐activated protein kinase
SARS‐CoV: Severe acute respiratory syndrome coronavirus
shRNA: Short‐hairpin Ribonucleic acid
SINE: Short INTerspersed Elements
SNP: Single‐nucleotide polymorphism

## Introduction: The *TP53* gene—a general homeostatic regulator

**Figure 1:**
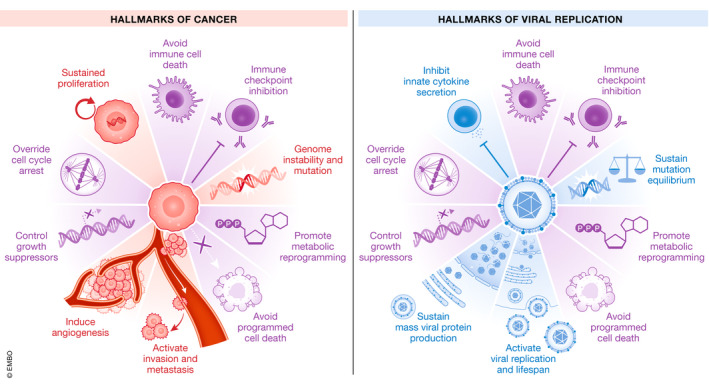
Schematic illustrating the similarities between the processes leading to cancer (Hallmarks of Cancer) and the processes involved in adapting to virus infection Similar hallmarks between cancer development and the cellular response to viral replication are shown in purple. These include avoiding immune cell death, immune checkpoint inhibition, promoting metabolic reprogramming, avoiding programmed cell death, overriding growth suppressors *(TP53)* and cell cycle arrest. Hallmarks specific to cancer and viral replication are represented in orange and blue respectively.

Homeostasis is a dynamic equilibrium in which continuous changes occur to maintain internal biochemical conditions for multicellular organisms to live and reproduce in an ever‐changing external environment. Any deviation from the limits of the internal conditions triggers a stress response that activates regulatory processes rapidly restoring the initial balance (feedback control) (Alfadda & Sallam, 2012; Marques *et al*, 2016; Horwitz *et al*, 2019). If homeostasis is successful, an animal survives; if unsuccessful, death ensues. However, conflicts can emerge between cellular and organismal fitness, so ensuring cooperation among cells is a major challenge in the evolution of complex organisms. For example, an individual cell within a tissue may gain a proliferation/fitness advantage by mutation or by better access to nutrients (close to blood vessels) and therefore outgrow the surrounding cells. This may compromise tissue homeostasis and eventually the survival of the animal. This is the case for changes that accrue over time that lead to cancer ((Hanahan & Weinberg, 2011), Fig 1). Similarly, cells and tissues must adapt to both acute and chronic infections and in so doing they acquire multiple changes, which are remarkably similar to those leading to cancer (Fig 1). Such changes suggest that there are common control mechanisms underpinning these adaptive processes. Over the past two decades, studies have identified that the *TP53* gene encodes a network of p53 proteins (p53 isoforms). Despite there being little mechanistic data, several lines of evidence suggest that the p53 network plays a central role in adaptive homeostasis by modulating and coordinating gene expression programmes that ensure cooperation among cells and tissues. In this review, we provide evidence of how different biological processes are regulated by the p53 network to maintain cellular and organismal homeostasis.

Evidence for the *TP53* network contributing to adaptive homeostasis comes from an extensive study aimed at identifying genes required to foster cell cooperation (Dejosez *et al*, 2013). A genome‐wide screen was carried out in murine‐induced pluripotent stem cells (iPSC) using an shRNA library of 150,000 target genes (Dejosez *et al*, 2013). They identified a small network of genes that cluster closely together and centre on *TP53*. This would be expected as p53 becomes activated (post‐translationally modified) by signals emitted from sensors in response to various stresses (e.g. DNA damage, oncogene activation, virus infection, oxidative stress, hypoxia), enabling it to facilitate cellular cooperation allowing cells to adapt to a changing environment in a co‐ordinated manner. As p53 is a transcription factor, these adaptations result in alterations to the transcriptional programme to maintain cellular homeostasis (reviewed in Braithwaite & Prives, 2006; Hafner *et al*, 2019). In addition, and consistent with p53 being important in cellular cooperation, phylogenetically, the *TP53* gene is found only in holozoa (Bartas *et al*, 2019), a clade of opisthokont eukaryotes that comprises the animals (moving multicellular organisms) and the motile unicellular organisms, choanoflagellates, Filasterea and Teretospore that demonstrate a degree of multicellularity (King *et al*, 2008).

A fundamental role for p53 in cell cooperation may also be inferred from the consequences of abnormal p53 activation during development. This induces congenital malformations, features of CHARGE syndrome: A disorder that affects many areas of the body (CHARGE: Coloboma, Heart defects, Atresia choanae (also known as choanal atresia), growth retardation, Genital abnormalities and Ear abnormalities) (Van Nostrand *et al*, 2014). Other pathologies from aberrant expression of p53 include premature ageing (Wu & Prives, 2018); neurodegeneration (Szybińska & Leśniak, 2017); diabetes (Kung & Murphy, 2016); cardiovascular diseases (Mak *et al*, 2017); chronic inflammation (Cooks *et al*, 2014); arthritis (Zhang *et al*, 2016) and susceptibility to infection. *TP53* is also by far the most frequently mutated gene in somatic cancer (Donehower *et al*, 2019) and germline mutations in *TP53* cause the inherited cancer predisposition disorder Li‐Fraumeni Syndrome (Nichols *et al*, 2001; Olivier *et al*, 2003; Guha & Malkin, 2017).

Thus, *TP53* plays a key role in cellular cooperativity and in multiple developmental processes to ensure normal tissue function and thus organismal homeostasis (Fig 1).

## p53 isoforms—a cooperative network of proteins

**Figure 2:**
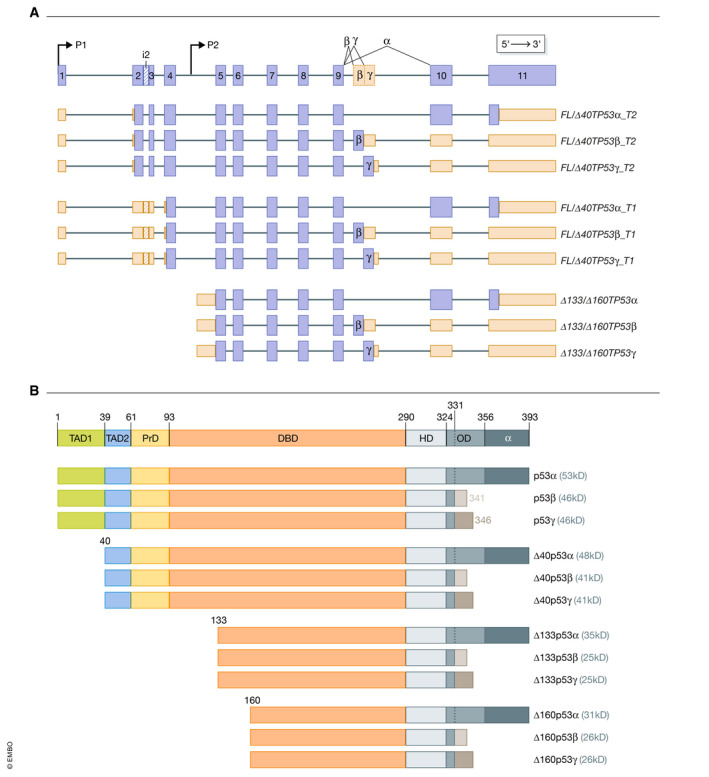
Structure of the *TP53* gene, encoded transcripts (A) and proteins (B) (A) Schematic demonstrating the *TP53* gene locus and the 9 *TP53* RNA transcripts known to be generated by alternative splicing and alternative promoter usage (P1 and P2). At the top of the figure, exons represented by blue boxes, including the regions the alternatively spliced transcripts α, β and γ variants. 5’UTR and 3’UTR are shown in orange. (B) Schematic of the canonical p53 protein and the 12 known isoforms. *TAD*1 Transactivation domain 1, *TAD*2 Transactivation domain 2, *PrD* Proline‐rich domain, *NLS* nuclear localization signal, *OD* Oligomerization domain.

To date, the human *TP53* gene expresses nine mRNAs (Fig 2A) giving rise to 12 proteins (Fig 2B) (Bourdon, 2014). The p53 protein isoforms are designated FLp53 or p53, Δ40p53, Δ133p53 and Δ160p53, each with C‐terminal alternative splice variants α, β and γ (Fig 2). p53 products are transcribed from the P1 promoter and use the first AUG in exon 2. Δ40p53 products are also transcribed from the P1 promoter and use an internal ribosome entry site (Bourdon *et al*, 2005). Transcription of the Δ133p53 and Δ160p53 products occurs from the P2 promoter in intron 4 (Marcel *et al*, 2010a, 2010b). The C‐terminal isoforms are generated by alternative splicing of intron 9, giving rise to exons 9β and 9γ, both of which contain stop codons preventing expression of exons 10 and 11 (Fig 2A).

A number of functional studies have shown that the isoforms have both overlapping and distinct functions with canonical p53α. Δ40p53α retains the second transactivation domain and the entire DNA‐binding domain, therefore, can transactivate many of the known p53α target genes (Hafsi *et al*, 2013) and other genes, including those involved in promoting cell differentiation (Ungewitter & Scrable, 2010). Δ133p53α contains most of the DNA‐binding domain and can directly bind to specific DNA sequences to transactivate genes (Chen *et al*, 2009; Gong *et al*, 2015). Δ133p53α can also bind to co‐factors that have DNA‐binding ability such as Early Growth Response Protein 1 (EGR1) (Xie *et al*, 2017), p63 (Marcel *et al*, 2012), ΔNp63 (Gong *et al*, 2018) and p73 (Marcel *et al*, 2012). Δ133p53β has been shown to transactivate a range of genes involved in cell proliferation, angiogenesis and immune regulation (Kazantseva *et al*, 2019). There is also evidence that under different conditions, these isoforms can function in concert (Fujita *et al*, 2009; Aoubala *et al*, 2011; Bernard *et al*, 2013) or in opposition to each other (Takahashi *et al*, 2014; Slatter *et al*, 2015; Horikawa *et al*, 2017; Gong *et al*, 2020). p53 isoforms contribute to many activities attributed to p53α, notably in cell cycle regulation and apoptosis. A comprehensive list of these contributing activities can be found in recent reviews (Joruiz & Bourdon, 2016; Kazantseva *et al*, 2018b; Anbarasan & Bourdon, 2019). The mechanistic basis underpinning cooperative activity probably involves hetero‐oligomerization of p53 isoforms either through the oligomerization or the DNA‐binding domains (Fig 2B). Hetero‐oligomers formed by a combination of p53 isoforms in response to multiple and often concomitant signals may have selective interactions with promoters and with the RNA polymerase II transcription machinery (Bourdon *et al*, 2005; Chen *et al*, 2009; Fujita *et al*, 2009; Meek & Anderson, 2009; Aoubala *et al*, 2011; Bernard *et al*, 2013; Hafsi *et al*, 2013; Marcel *et al*, 2014; Gong *et al*, 2015). This flexibility of interactions is facilitated by the modular nature of the p53 isoforms conferred by differing N‐terminal and C‐terminal domains. These allow a broad repertoire of signals that can be integrated by p53 isoforms, fine‐tuning the specificity of interactions. In addition, as p53 binds specifically with high affinity to different DNA sequences (p53 Response Elements, p53REs), which are also degenerate, the repertoire of transcriptional target genes is extended. It is estimated that there are 23,808 different ways to generate a high affinity p53RE (Khoury & Bourdon, 2011). As well as the affinity and specificity of p53 proteins for the different p53RE sequences, they are also influenced by their number, as most p53‐inducible genes contain clusters of p53REs separated by 0–13 bp. This enables oligomerization and stacking of p53 proteins on DNA (Kern *et al*, 1991; Stenger *et al*, 1994; Bourdon *et al*, 1997; Vyas *et al*, 2017; Lei *et al*, 2019; Ly *et al*, 2020; Senitzki *et al*, 2021). Thus, the large variety of p53 REs and permutation of p53 isoforms allow the p53 network to regulate the expression of a broad range of genes to maintain and restore cell and organ function and thus organismal integrity (Beno *et al*, 2011; Afek *et al*, 2020; Farkas *et al*, 2021). This would not be possible if *TP53* encoded a single protein product.

Over the past decade, using diverse human cell lines and animal models, data have consistently demonstrated that the balance of expression levels of between p53 isoforms ultimately defines the p53‐mediated cell responses to different and often simultaneous input signals (reviewed in Joruiz & Bourdon, 2016). Similar to p53α, dysregulation of p53 isoforms has been implicated in multiple pathologies. Using animal models, including zebrafish (Chen *et al*, 2005, 2009; Davidson *et al*, 2010; Elabd *et al*, 2019; Ye *et al*, 2020), drosophila (Jassim *et al*, 2003; Dichtel‐Danjoy *et al*, 2013; Kashio *et al*, 2014; Simón *et al*, 2014), pigs (Niu *et al*, 2021) and mice (Maier *et al*, 2004; Hinault *et al*, 2011; Slatter *et al*, 2011; Hamard *et al*, 2013; Senturk *et al*, 2014; Campbell *et al*, 2018; Kazantseva *et al*, 2018b), experiments have shown that aberrant expression of the isoforms leads to embryo malformation (Davidson *et al*, 2010) and other pathologies. These include premature ageing (Maier *et al*, 2004; Davidson *et al*, 2010; von Muhlinen *et al*, 2018); neurodegeneration (Medrano *et al*, 2009; Turnquist *et al*, 2016); diabetes (Hinault *et al*, 2011); cardiovascular diseases (Ye *et al*, 2020); chronic inflammation (Slatter *et al*, 2011; Campbell *et al*, 2012, 2018; Roth *et al*, 2016; Kazantseva *et al*, 2018a, 2018b, 2019; Mehta *et al*, 2018); impaired immune responsiveness (Mondal *et al*, 2013; Gong *et al*, 2015, 2016a, 2016b, 2020) and cancer (reviewed in Kazantseva *et al,* 2018b); Vieler & Sanyal 2018). Thus, not only p53α but also the p53 network in general initiates adaptive responses at multiple levels to ensure organismal homeostasis (Fig 1).

## p53, isoforms, viruses and other pathogens

**Figure 3:**
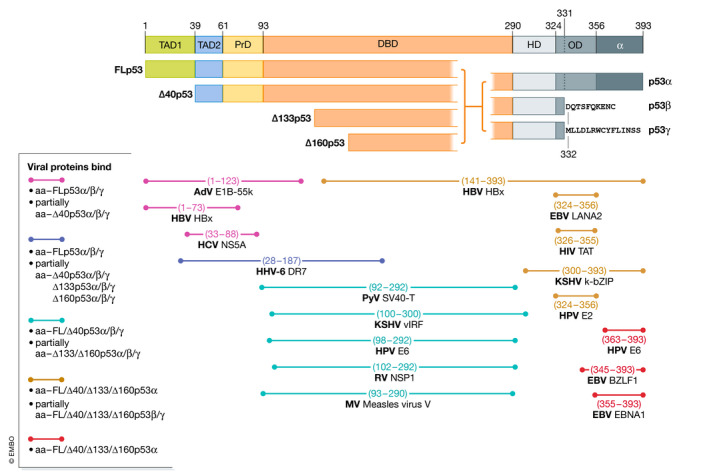
Map illustrating regions on p53 protein that are bound by viral proteins post infection Schematic of the canonical p53 protein and the 12 known isoforms. *TAD*1 Transactivation domain 1, *TAD*2 Transactivation domain 2, *PrD* Proline‐rich domain, *NLS* nuclear localization signal, *OD* Oligomerization domain. The horizontal bars at the bottom show the amino acids (aa) bound by viral proteins on p53 and the overlap with potential p53 protein isoforms.

Arguably, one of the most profound exogenous sources of both cellular and organismal homeostatic imbalance is infection by viruses and other pathogens. Cells need to respond to the stresses of virus replication and whole organisms to the consequences of viraemia (Fig 1). Indeed, many of the common stresses known to activate p53 are consequences of infection. p53α was discovered as a protein in complex with SV40 large tumour (LT) antigen (Lane & Crawford, 1979) and the adenovirus (Ad) E1b55 kD protein (Linzer & Levine, 1979). Since then, the list of viruses and viral proteins that interact with (at least) p53α has grown very extensively (Table 1) and now includes other DNA viruses such as Epstein–Barr Virus (EBV, reviewed in Chatterjee *et al,* 2019), human papilloma viruses (HPV) (Parish *et al*, 2006) and herpesviruses (Maruzuru *et al*, 2013), but also RNA viruses from many taxonomic groups. These include flaviviruses, retroviruses, influenza viruses, parvoviruses and coronaviruses (Fig 3; Table 1; reviewed in Aloni‐Grinstein *et al*, 2018) all of which affect p53 function. It is interesting that most of these viral proteins bind to p53α in the DNA‐binding domain (present in all 12 isoforms) and the C terminus (Fig 3). Moreover, several of these proteins bind regions of p53 encompassed by p53β/γ splice, suggesting that one or more p53 isoforms also interact with viral proteins. Given such diversity of viruses with different tissue tropisms and modes of replication, it seems reasonable to suggest that the evolution of the *TP53* gene network has occurred in adapting to the many stresses imposed by the large variety of viruses and pathogenic microorganisms.

**Table 1:** Interaction of viral proteins with p53 and their consequences.

DNA/RNA virus	Name of virus	Viral protein	Interaction with p53/p53 isoforms	Consequence of p53/viral protein interaction	Ref
Double‐stranded DNA virus	SV40 (John Cunningham BK virus)	T antigen	T antigen interacts with p53 and alters its ability to transactivate target genes	T antigen inhibits p53 activity Agno protein promotes p53 activity	Lane and Crawford (1979); Jenkins *et al* (1988); Jiang *et al* (1993); Darbinyan *et al* (2002)
		Agno protein	Agno protein enhances the transactivation of p53 target genes		
	High Risk Human Papillomavirus (HPV) Oncogenic	High‐risk HPV E2	Interacts with p53 and induces transactivation of p53 target genes	E2 and E7 activate p53 E6 inhibits p53 activity	Seavey *et al* (1999); Parish *et al* (2006); Bernard *et al* (2011)
		High‐risk HPV E6	Associated with E6AP and targets p53 for proteasome degradation		
		High‐risk HPV E7	Increase p53 stability		
	Adenovirus (Oncogenic)	E1A	E1A inhibits proteasomal degradation of p53. E1A also promotes the activation of p53 target genes	E1A activates p53 E1B‐55K and E4orf6 inhibit p53	Linzer and Levine (1979); Braithwaite *et al* (1990); Lowe and Ruley (1993); Nevels *et al* (1997); Martin and Berk (1998); Nakajima *et al* (1998); Royds *et al* (2006)
		E1B‐55 kDa	Inhibits the activation of p53 target genes. In combination with E4orf6 promotes p53 degradation		
	Simplex virus HSV‐1 or HSV‐2 (non‐oncogenic)	ICP0	Promotes proteasomal degradation of p53	ICP0 inhibits p53 ICP4 promotes p53 stability	Boutell and Everett (2003, 2004)
		ICP4	Promotes stabilization of p53 via post‐translational modification		
	Cytomegalovirus (CMV non‐oncogenic)	IE1‐72	Interacts with p53 and downregulates the activation of downstream targets.	IE1‐72 and IE2‐86 alter the activation of p53 target genes. UL84 promotes p53 stability	Hwang *et al* (2009)
		IE2‐86	Interacts with p53 and alters transactivation of p53 targets.		
		UL84	Interacts with and stabilizes p53		
	Human Herpes virus 6 (non‐oncogenic/oncogenic)	HHV‐6 U14	Alters cellular localization of p53 from the cytoplasm to the nucleus.	U14 promotes p53 activity ORF‐1 inhibits p53 activity	Kashanchi *et al* (1997); Takemoto *et al* (2005)
		ORF‐1 (DR7)	Inhibits the activation of p53 target genes		
	Epstein–Barr Virus (EBV) (non‐oncogenic/oncogenic)	BZLF‐1	Interacts with p53 and alters transactivation of p53 targets.	BZLF‐1, EBNA3C and LMP‐1 alter p53 activity. EBNA1 inhibits p53	Chatterjee *et al* (2019)
		EBNA1	Promotes proteasomal degradation of p53 by activating USP7.		
		EBNA3C	Alters p53 transcriptional activity either by direct interaction or via modulation of Gemim3		
		LMP‐1	Inhibits transcriptional activity of p53 indirectly via NF‐kB pathway, IRF5 or stimulation of A20 expression		
	Kaposi Sarcoma‐Associated Herpes virus (KSHV) (oncogenic)	LANA1 and LANA2	Interact with p53 and inhibit its transcriptional activity	K8β activates p53 LANA1/2, vIRF1/3/4 and k‐bZIP inhibit p53 activity	Friborg *et al* (1999); Yamanegi *et al* (2005); Lee *et al* (2009); Chen *et al* (2010); Baresova *et al* (2014); Chavoshi *et al* (2016)
		vIRF1, vIRF3, vIRF4	Suppress p53‐dependent transcription and apoptosis		
		k‐bZIP	Binds the C terminus of p53 and alters p53 transcriptional activity. Recruits p53 to PML bodies.		
		K8β	Antagonizes k‐bZIP and induces p53 and p21 activity		
	Vaccinia Virus (non‐oncogenic)	B1R kinase	MDM2‐dependent degradation of p53 despite phosphorylation at Thr18. Hyperphosphorylate p53 at Ser15 and Thr18. Alters p53 transcriptional activity	Inhibits p53 activity	Lopez‐Borges and Lazo (2000); Barcia *et al* (2002); Santos *et al* (2004)
Partial double/single‐stranded DNA Virus	Hepatitis B (oncogenic)	HBx	Interacts with C terminus of p53 and the ratio of HBx:p53 alters the transcriptional activity of p53.	The ratio of HBx:p53 impacts p53 activity	Truant *et al* (1995); Wang *et al* (1995); Lee and Rho (2000)
Double‐stranded RNA virus	Rotavirus (non‐oncogenic)	NSP1	Initial infection: interacts with DNA‐binding domain of p53, resulting in ubiquitination and degradation of p53. Late infection: NSP1‐p53 interaction is reduced by an unknown mechanism resulting in stabilization of p53.	NSP1 inhibits p53 during the initial phase of the infection.	Bhowmick *et al* (2013)
	Avian Reovirus (ARV)	ARV σC	Increases p53 mRNA and protein expression. Promotes p53 phosphorylation at Ser46 and Ser392.	Activates p53	Ping‐Yuan *et al* (2006); Chulu *et al* (2007)
(+) Single‐stranded RNA virus	Enteroviruses Poliovirus (non‐oncogenic)	Viral encoded protease 3C(Pro)	Recruits p53 to PML nuclear bodies Targets p53 for degradation	Inhibits p53 activity	Weidman *et al* (2001)
	Flavivirus Dengue Virus (non‐oncogenic)	DENV2	DENV2 upregulates p53‐2 (p53 paralogue in mosquitoes)	Activates p53	Chen *et al* (2018)
	Flavivirus Zika Virus (non‐oncogenic)	ZIKV‐Env	Promotes phosphorylation of p53 at Ser15 and increases p53 levels during ZIKV infection	Activates p53	Ghouzzi *et al* (2016)
	Flavivirus West Nile Virus (non‐oncogenic)	WNVCp	Promotes p53 stabilization	Activates p53	Yang *et al* (2008)
	Hepatitis C Virus (non‐oncogenic/oncogenic)	NS5A and NS3	Directly binds with the C terminus of p53 and prevents its transcriptional activity. Alters post‐translational modification of p53. Enhances MDM2‐mediated proteasomal degradation of p53.	NS5A and NS3: Low levels activate p53 while high levels inhibit p53.	Otsuka *et al* (2000); Lan *et al* (2002); Deng *et al* (2006); Bittar *et al* (2013)
		NS2	Modulates p53 function by altering the cellular localization of p53	NS2: Inhibits p53 activity.	
	Coronavirus SARS‐CoV (COVID‐19) MERS (non‐oncogenic)	SUD	Increases ubiquitin‐mediated degradation of p53	SARS‐CoV‐infected cells degrade p53. SARS‐CoV‐infected cells express the C‐terminal alternative splice variant of the p53.	Leong *et al* (2005); Ma‐Lauer *et al* (2016); Xiong *et al* (2020)
		PL(pro)	Increases ubiquitin‐mediated degradation of p53		
		PLP2 (HCoV‐NL63)	Increases ubiquitin‐mediated degradation of p53		
(−) Single‐stranded RNA virus	Orthomyxoviridae Influenza (non‐oncogenic)	IAV	Activates p53. Alters cellular localization of p53 Results in downregulation of host p53 pathways.	p53 is elevated at the beginning of infection and during the middle‐late stage of infection.	Terrier *et al* (2012); Dubois *et al* (2019)
		NS1	Alters p53 splicing in combination with CPSF4. It favours the beta and gamma spice variants Inhibits p53 transcriptional activity. Targets MDM2 and thus contributes towards p53 stability. Facilitates phosphorylation of p53 at ser46 and ser37 contributing to apoptosis		
	Respiratory Syncytial Virus (non‐oncogenic)	NS1 and NS2	Inhibit p53 activity by promoting proteasome‐dependent p53 degradation at late stages of infection.	Modulates p53 activity	Bian *et al* (2012); Machado *et al* (2018)
		RSV‐M	RSV‐M induced p53 and p32 accumulation to induce cell cycle arrest		
	Parainfluenza virus (non‐oncogenic)	dsRNA	Presence of dsRNA can trigger downregulation of p53	Modulates p53 activity	Marques *et al* (2005)
	Measles virus (non‐oncogenic)	Measles virus V	Directly interacts with DNA‐binding domain of p53 and its family member p73 to delay apoptosis	Modulates p53 and p73 activity	Cruz *et al* (2006)
Single stranded RNA‐Retrovirus	HIV‐1 and HIV‐2 (non‐oncogenic)	Tat	Inhibits activation of p53 target genes, either by repressing the p53 promoter, directly binding or altering the acetylation status of p53 at Lys320.	Early infection inhibits p53 activity. Late infection activates p53.	Li *et al* (1995b); Greenway *et al* (2002); Harrod *et al* (2003); Amini *et al* (2004); Perfettini *et al* (2005); Ali *et al* (2020)
		Nef	Directly binds p53 and prevents its transcriptional activity. It also facilitates a reduction in the half‐life of p53 protein		
		Vpr	Forms a ternary complex with p53 and Sp1 which enhances p21^WAF1/CIP1^ expression.		
		Env	Enhances p53 phosphorylation at Ser15		
	Human T‐lymphotropic virus (HTLV) (oncogenic)	Tax	Stabilizes p53 but alters its transcriptional activity by the repression of phosphorylation at Ser15 and Ser392	Modulates p53 activity to induce cell cycle arrest and prevent apoptosis	Pise‐Masison *et al* (2000)

A survey of viruses suggests that p53α is often targeted by viral proteins having evolved strategies to promote degradation, sequestration or to inhibit the transactivation capability of p53α (see detailed documentation in Table 1). To sustain mass viral protein production, viruses target p53 in infected cells to override cell cycle control, promote metabolic reprogramming (Frese *et al*, 2003; Yu *et al*, 2011; Ramière *et al*, 2014; Kindrachuk *et al*, 2015; Bilz *et al*, 2018; Choi *et al*, 2020; Lacroix *et al*, 2020; Singh *et al*, 2020) and prevent premature cell death via apoptosis (reviewed in Fan *et al*, 2018). Additionally, to prevent destruction, infected cells also over‐ride multiple components of the immune response including recruitment and activation of immune cells (Chua *et al*, 2014; Menendez *et al*, 2019), cytokine secretion (Machado *et al*, 2018), processing and presentation of viral peptides on the cell surface (Herzer *et al*, 2003; Wang *et al*, 2013). For an efficient productive infection to occur, it is necessary for viruses to abolish all these antiviral defence systems. If the virus fails to inactivate simultaneously all components of the antiviral defence system, a productive infection cannot proceed. By manipulating p53, viruses can control all the different systems simultaneously, emphasizing the adaptive nature of the p53 response. Thus, as might be expected, loss of the *TP53* gene or reduced overall expression of p53 protein leads to marked increases in the yields of several viruses (Lu *et al*, 1999; Balachandran *et al*, 2001; Farley *et al*, 2004; Pampin *et al*, 2006; Wright & Leppard, 2013). Some examples of the integral nature of the p53 network in virus life cycles are discussed below.

A topical example of the p53 network influencing virus replication is with coronaviruses (Ma‐Lauer *et al*, 2016). SARS‐CoV and other coronaviruses were found to be severely inhibited in cells expressing p53α. To circumvent p53α, the viral non‐structural protein 3 stabilizes host Ring Finger and CHY Zinc Finger Domain Containing 1 (RCHY1) protein, an E3 ubiquitin ligase that binds and promotes p53α degradation. Of interest, RNA‐seq analysis of PBMCs from SARS‐CoV‐2 patients showed an increase in *TP53* signalling (Xiong *et al*, 2020). A number of the p53 isoforms have been implicated in modulating immune and inflammatory responses (see below), and increased mRNAs from pro‐inflammatory genes were also evident in samples from infected patients (Xiong *et al*, 2020). Thus, we speculate that changes in the levels of the p53 isoforms may contribute towards the immunopathology of SARS‐CoV‐2 infection.

In another example, p53 and isoforms have a major impact on influenza A virus (IAV). p53α inhibits IAV replication in cell culture (Terrier *et al*, 2012) and p53 null mice have more viraemia and lung pathology than control mice (Yan *et al*, 2015). However, when p53α is co‐expressed with p53β, p53α no longer inhibits IAV replication (Terrier *et al*, 2012). Co‐expression of p53α with Δ133p53α increases IAV replication by ˜ 200 fold. Thus, the relative levels of the p53 isoforms appear to dictate the outcome of an IAV infection. In a separate study, lung cancer cells overexpressing Δ40p53 showed that Δ40p53 inhibited interferon‐induced transmembrane protein (IFITM) expression making the cells highly susceptible to IAV infection (Wang *et al*, 2018). In addition, recent data have shown that IAV non‐structural (NS1) protein and CPSF4 (cellular protein cleavage and polyadenylation specificity factor 4) interact to promote *TP53* splicing to generate p53β/γ, which together promote IAV replication (Dubois *et al*, 2019). Other IAV proteins NS5A and NS3 decrease p53α activity by counteracting the Protein Kinase R pathway and subsequently phosphorylating eukaryotic translation initiation factor 2‐alpha (eIF2α), which protects against viral infection (Gong *et al*, 2004; Majumder *et al*, 2001). Of interest, eIF2α has been implicated in promoting translation of Δ40p53 (Bourougaa *et al*, 2010). These data indicate an interplay between p53 isoforms and viral proteins in regulating virus replication.

p53 proteins may also be important in the adenovirus life cycle. Two independent studies have showed that p53 enhanced adenovirus replication by increasing expression of late‐viral genes (Royds *et al*, 2006; Wright & Leppard, 2013), despite p53 being degraded by a complex of E1b55 kD and E4orfE6 protein during the early phase of infection ((Querido *et al*, 2001); Table 1).

p53 proteins also play key roles in the replication of retroviruses, such as Human Immunodeficiency Virus (HIV‐1). Once the virus enters a cell, the viral RNA genome is reverse‐transcribed making several copies of linear double‐strand DNA that insert into the genome of the host cells (i.e. provirus). This creates DNA breaks which activates p53 (Takaoka *et al*, 2003). At each end of the viral genome are long terminal repeats (LTR) sequences. These LTRs harbour multiple DNA‐binding sites for transcription factors and chromatin remodelling proteins that are required for the regulation of viral RNA synthesis and the initiation and termination of transcription. Early studies reported that HIV‐1 LTRs contain p53REs and that p53 can modulate HIV‐1 LTR transcriptional activity, directly and indirectly by interacting with other transcription factors (Gualberto & Baldwin, 1995; Gualberto *et al*, 1995; Bargonetti *et al*, 1997). p53 suppresses Tat activity, a major transactivator of HIV‐1 LTR, which in turn restricts HIV replication (Li *et al*, 1995a). p53 also inhibits reverse transcription of HIV‐1 in non‐cycling cells through the induction of the p53‐regulated gene *CDKN1A* (encoding the cyclin‐dependent kinase inhibitor p21) and *SAMHD1* (encoding a deoxyribonucleotide triphosphate (dNTP) triphosphohydrolase) which limits the intracellular pool of dNTP thus inhibiting reverse‐transcriptase activity (Shi *et al*, 2018). In addition, reducing p53 by increasing MDM2 levels was shown to facilitate early HIV‐1 replication (Breton *et al*, 2019). Recently, the impact of the different p53 isoforms on HIV‐1 replication in macrophages was investigated (Breton *et al*, 2021). Δ133p53 was found to increase HIV‐1 replication by promoting phosphorylation and inactivation of SAMHD1. Conversely, p53β inhibited viral production. Thus, as is the case with IAV, the relative balance of p53 isoform level influences the outcome of the p53‐mediated anti‐HIV‐1 response.

During evolution, numerous retroviruses have integrated into the genome of animals and humans and the p53 network has evolved alongside to regulate transcription from these integrated sequences. About half of the human genome consists of DNA sequences derived from ancient viral infections (Lander *et al*, 2001; Venter *et al*, 2001; Hancks & Kazazian, 2016; Kazazian & Moran, 2017; Payer & Burns, 2019). These include Endogenous RetroViruses (ERVs), Long INterspersed Elements (LINEs) and Short INterspersed Elements (SINEs), which are collectively known as transposable elements (TEs). TEs include long terminal repeats (LTRs) and encode their own reverse transcriptase and are transcribed by RNA Polymerase II (Lander *et al*, 2001). They contain transcription initiation sites, splice sites, polyadenylation sites and multiple transcription factor‐binding sites, including for p53 (Cui *et al*, 2011; Hancks & Kazazian, 2016; Kazazian & Moran, 2017; Payer & Burns, 2019). TEs can also behave as enhancers and silencers, facilitate chromatin modelling and promote chromosome rearrangements (Cui *et al*, 2011; Hancks & Kazazian, 2016; Kazazian & Moran, 2017; Payer & Burns, 2019). The millions of TEs dispersed throughout the genome have contributed to evolution by providing an abundant source of novel protein coding and DNA regulatory sequences (Cui *et al*, 2011; Hancks & Kazazian, 2016; Kazazian & Moran, 2017; Payer & Burns, 2019). Several studies have identified p53REs in the 5’UTRs of these TEs and that p53 can facilitate long distance transcriptional regulation either directly or via inducing transcription of enhancer RNAs (eRNAs). These are important for maintaining an open chromatin state readily accessible to transcription factors and cofactors and are cell‐lineage specific. eRNAs function in *cis* to contribute to the dynamic stabilization of enhancer–promoter looping and in *trans* to regulate chromatin‐remodelling (Melo *et al*, 2013; Allen *et al*, 2014; Léveillé *et al*, 2015; Sartorelli & Lauberth, 2020). Interestingly, it has been demonstrated that p53α homotetramers induced eRNA transcription and G_1_ cell cycle arrest upon treatment of MCF10A cells with the p53 agonist, nutlin (Levandowski *et al*, 2021). In contrast, Δ40p53α:p53α heterotetramers inhibited eRNA transcription and increased transcription of genes essential for cell cycle progression, including those in the E2F, mTOR and IGF‐1 signalling pathways. This study demonstrates that the combination of p53 isoforms can influence the expression of TEs and eRNAs, altering the response to stress.

TE‐derived *cis‐*regulatory sequences also enable p53 to have chromatin pioneering activity that allows p53 to initiate novel gene expression programmes (Sammons *et al*, 2015; Yu & Buck, 2019). This could include antiviral and tumour‐suppressive activities or oncogenic and anti‐inflammatory activities or the generation of neo‐antigens (Levine *et al*, 2016; Wylie *et al*, 2016; Buzdin *et al*, 2017; Lemaître *et al*, 2017; Garcia‐Montojo *et al*, 2018; Grandi & Tramontano, 2018; Tiwari *et al*, 2018; Xue *et al*, 2020; Jansz & Faulkner, 2021).

Thus, the large variety of TE‐derived sequences provides a pool of potential new genes that allow organisms to adapt to many different environmental conditions. This, combined with the remarkable diversity of p53 regulatory capabilities through p53RE sequence diversity and the isoform network, that has evolved along‐side the TEs, provides a mechanism that allows p53 to trigger tailored adaptive responses to many environmental conditions, especially virus infections. In this way, p53 ensures the maintenance of organismal homeostasis.

Besides mammalian studies, p53 isoforms have also been found in the shrimp species *Litopenaeus vannamei* (Li *et al*, 2017) where they have been shown to affect not only virus replication but also the host response to infection. Two *TP53* transcripts have been identified in *L*. *vannamei*—the first being LvFLp53 which corresponds to human p53α, whilst the second, LvΔNp53, lacking the N‐terminal 145 amino acids, corresponds to human Δ133p53α. Similar to human Δ133p53, LvΔNp53 is transcribed from an internal promoter in intron 3. LvFLp53 was found to inhibit the replication of white spot syndrome virus (WSSV) whilst the replication of WSSV was enhanced by LvΔNp53. Silencing of LvFLp53 increased WSSV load and higher shrimp mortality. In addition, LvFLp53 downregulated the pro‐inflammatory nuclear factor kappa B (NF‐κB) pathway, but LvΔNp53 increased NF‐κB signalling. Thus, the relative combination of p53 isoforms in the shrimp, as with IAV in mice, has a marked influence on the outcome of virus infection affecting both virus replication and adaptive immunity of the host.

In addition to viruses, bacteria have been found to target and inactivate p53α. Cells infected with various species of *Chlamydia* result in induction of MDM2 (González *et al*, 2014). MDM2 is an E3 ubiquitin ligase that antagonizes p53 and causes proteasomal degradation of p53 through the activation of the MDM2‐p53 axis (González *et al*, 2014). Another example of p53 modulation is from *Shigella flexneri*. Infection results in an early induction of p53α, but the p53 response and cell death are impaired by virulence effector VirA‐induced calpain protease activity that causes amino terminal cleavage of p53α (Bergounioux *et al*, 2012). Similarly, the intracellular bacterial pathogen *Neisseria gonorrhoeae* suppresses p53 post infection of epithelial cells (Vielfort *et al*, 2013) and *Salmonella typhimurium* modulates p53 activity to favour Salmonella colonization (Wu *et al*, 2010). *Helicobacter pylori* (*H. pylori*) activates AKT in human gastric epithelial cells, which in turn results in phosphorylation and activation of MDM2 and subsequent inactivation of p53α (Wei *et al*, 2010). However, *H. pylori* also induces expression of Δ133p53 and Δ160p53 isoforms (Wei *et al*, 2012) in gastric epithelial cells and similarly, in Mongolian gerbil, *H. pylori* infection results in an induction of Δ153p53 mRNA (orthologous to human Δ133p53/Δ160p53) (Wei *et al*, 2012). Increased Δ133p53 isoform in turn increases NF‐κB activity and the mRNA expression of multiple downstream target genes including IL‐6, Bcl‐2 and IL‐8 (Wei *et al*, 2012). Thus, as for viruses and several bacterial species, modulating the p53 network appears to be an integral part of the infection process and also initiating adaptive responses to ensure host survival (Fig 1).

## p53, isoforms and oxidative stress

Another trigger leading to loss of homeostasis that occurs in response to pathogen infection and inflammation are reactive oxygen species (ROS) (Alfadda & Sallam, 2012). ROS are generated during mitochondrial oxidative phosphorylation. Oxidative stress occurs when there is an excessive accumulation of ROS within the cell (Sies & Jones, 2020). Oxidative stress results in macromolecular damage leading to aberrant intracellular signals to promote cell proliferation and survival at subtoxic levels, while at higher levels cause cell death or senescence (reviewed in Ray *et al*, 2012, Fig 1). It is well known that ROS activate p53 and evidence suggests that p53 isoforms define the cell response to ROS (Liu *et al*, 2008). ROS increases p53 and the 20S proteasome resulting in p53α cleavage leading to enhanced expression of Δ40p53, which in turn regulates p53α’s transcriptional activity (Solomon *et al*, 2017). High ROS environments result in mitochondrial DNA damage, which can be repaired by DNA pol γ which interacts with p53α. A recent study demonstrated that both Δ40p53 and Δ133p53 are present in the mitochondria and both of these interact with p53α to form dimers and tetramers (Liu *et al*, 2017a). Interestingly, *in vitro* studies showed that p53α and Δ40p53 enhanced the activity of mitochondrial DNA pol γ while Δ133p53 inhibited the activity of DNA pol γ (Liu *et al*, 2017a). Mouse embryonic fibroblasts (MEF) from Δ122p53 mice, a transgenic mouse model of Δ133p53 (Slatter *et al*, 2011), were also resistant to oxidative stress (Kazantseva *et al*, 2018b). Similarly, liver epithelial cells treated with oxidative stress resulted in induction of Δ133p53 expression which protected cells from DNA damage and facilitated their survival (Gong *et al*, 2016b). Finally, mouse cells expressing p53Ψ, a unique p53 isoform generated due to an alternative 3’ splice site in intron 6, increased mitochondrial pore permeability and ROS necessary for epithelial to mesenchymal transition (Senturk *et al*, 2014). Thus, p53 isoforms may determine the impact ROS has on cellular functions and homeostasis. Of note, p53Ψ is not physiologically expressed in human cells. The human *TP53* gene can only express p53Ψ as a result of mutations at the intron 6/exon 7 boundary acceptor splicing site (Senturk *et al*, 2014).

## p53, isoforms and cellular senescence

Cellular senescence refers to a state of permanent proliferative arrest. It is a stress response aiming to preserve cellular and organismal homeostasis. Pathogens are known to exploit ageing cells and are able to promote persistent induction of senescence resulting in loss of homeostasis (Humphreys *et al*, 2020). Other senescent triggers include telomere attrition, oxidative stress, activated oncogenes and failure to repair DNA damage (reviewed in Vasileiou *et al*, 2019, Fig 1). Senescent cells do not proliferate but are metabolically active and are known for their increased secretory activity. Senescent cells carry out a complex pro‐inflammatory response known as senescence‐associated secretory phenotype (SASP), involving secretion of multiple cytokines and chemokines including IL‐1β, IL‐6, IL‐8 and CCL2‐5 which are mediated via NF‐kB signalling. Short‐term accumulation of senescent cells can be beneficial; however, chronic persistence can result in ageing and age‐related pathologies (Vasileiou *et al*, 2019). Two extensively studied pathways involved in the regulation of cellular senescence include p53/p21^cip1^ (inhibits cyclin/CDK complexes) and p16^INK4A^/Rb (CDK4 inhibitor/Retinoblastoma protein). Recent evidence suggests that p53 isoforms play an important role in cellular senescence, with the levels of p53, along with the duration and intensity of the stress, determining cell fate outcome (reviewed in Mijit *et al*, 2020). Evidence from multiple studies suggest that decreased levels of Δ133p53 and increased p53β are associated with promotion of cellular senescence (Gong *et al*, 2016a; Turnquist *et al*, 2016, 2019; Horikawa *et al*, 2017; von Muhlinen *et al*, 2018). Senescent CD8^+^ T lymphocytes are associated with increased p53β expression and loss of Δ133p53 expression. Restoration of Δ133p53 expression in near senescent CD8+ T cells resulted in rescue from the senescent phenotype and extended replicative lifespan (Mondal *et al*, 2013). Similarly, near‐senescent primary fibroblasts derived from Hutchinson–Gilford progeria syndrome (HGPS) patients exhibited low levels of Δ133p53 and high levels of p53β, while restoration of Δ133p53 expression resulted in delaying senescence and promoting proliferation as well as repair of DNA‐double strand breaks (von Muhlinen *et al*, 2018). Increased levels of p53β and decreased levels of Δ133p53 were also observed in colon adenomas with a senescent phenotype (Fujita *et al*, 2009). Importantly, TCR‐engineered CD8+ T cells transduced with Δ133p53α acquire a long‐term proliferative capacity, show superior cytokine secretion and enhanced tumour‐specific killing *in vitro* and in a mouse tumour model (Legscha *et al*, 2021). Taken together, these studies suggest that Δ133p53 is an integral component of the self‐renewal process in human somatic cells.

In addition to Δ133p53 and p53β, Δ40p53 may also play a role in senescence induction. MEF cells from mice expressing p44 (mouse orthologue of Δ40p53) showed reduced proliferative capacity and were positive for senescence‐associated‐β‐galactosidase, a marker of senescent cells (Gambino *et al*, 2013). In melanoma cells, expression of Δ40p53α was shown to inhibit the transcription of genes required for apoptosis induction including p21 and p53 upregulated modulator of apoptosis (PUMA) (Avery‐Kiejda *et al*, 2008). In contrast, p53β increased the transcription of these genes in melanoma cells (Avery‐Kiejda *et al*, 2008). However, in hepatocellular carcinoma (HCC) cells, Δ40p53α was associated with a higher proportion of senescent cells (Ota *et al*, 2017). Melanoma, glioblastoma, melanocytes and fibroblast cells expressing a lentivirus encoding Δ40p53α resulted in increased levels of activated p53α and apoptosis in the presence of proteotoxic stress (Takahashi *et al*, 2014). These seemingly contradictory results may be explained by alterations in the relative levels of Δ40p53 and other p53 isoform levels that coordinate the transcriptional activity of p53‐regulated promoters thereby affecting cellular homeostasis (Hafsi *et al*, 2013).

## p53 isoforms and regeneration

To ensure that homeostasis is restored in cells after stress, most tissues and organs undergo partial or complete regeneration. Cellular processes such as proliferation, apoptosis, differentiation and senescence occur at different stages of regeneration, which include inflammation, tissue reconstruction and remodelling; all processes known to involve p53 and isoforms.

There is evidence that p53α activities are differentially regulated during the initial phases of regeneration and at the differentiation phase (Antoniades *et al*, 1994; Wells *et al*, 2006; Pearson & Sánchez Alvarado, 2010; Yun *et al*, 2013; Charruyer *et al*, 2021). Δ40p53 and Δ133p53α play key roles in this process. Mouse embryonic stem cells (ESC) express high levels of p44 compared to other mouse tissues (Ungewitter & Scrable, 2010), highlighting its importance during development. Ectopic expression of Δ40p53 was found to enhance the proliferative capacity of ESC by maintaining pluripotency but reducing Δ40p53 levels caused cells to differentiate. Thus, Δ40p53 controls the switch from pluripotency to differentiation. This occurs by Δ40p53 modifying the activity of p53α to activate differentiation genes including Oct4, Nanog and the IGF‐1 receptor (Ungewitter & Scrable, 2010). Thus, the relative amounts of p53α and Δ40p53 dictate the biological outcome. In contrast to ESCs, transgenic mice homozygous for the Δ40p53 isoform have a shorter lifespan, reduced cell proliferation capacity and exhibit multiple premature ageing phenotypes, and again, this is linked to IGF‐1 signalling (Maier *et al*, 2004). Thus, Δ40p53 functions differently in a different cell context. Indeed, increased levels of p44 resulted in neuronal cell paraptosis and autophagy‐like cell death, contributing to neurodegeneration, which is also dependent on IGF‐1 signalling (Pehar *et al*, 2010). Collectively these data implicate Δ40p53 as an integral regulator of tissue regeneration through IGF‐1 signalling.

Co‐transfection of Δ133p53α with the Yamanaka factors in human fibroblasts significantly enhanced their reprogramming to induce pluripotent stem cells (iPSCs). The Δ133p53‐iPSC had a normal karyotype, stable microsatellite repeats and wild‐type mitochondrial DNA (Horikawa *et al*, 2017; Mondal *et al*, 2018). In contrast, iPSC generated from silencing p53α had significant mutations and eventually formed malignant tumours. Similarly, a separate study demonstrated that overexpression of Δ133p53 in iPSC resulted in inhibition of apoptosis, promoted DNA DSB repair foci resulting in a decrease in chromosomal aberration and an increase in reprogramming efficiency (Gong *et al*, 2016a).

In Zebrafish, the heart is able to be fully regenerated after amputation of up to 20% of the ventricle (Jopling *et al*, 2010). A recent study showed that expression of Δ113p53 (orthologue of Δ133p53) is activated in stressed cardiomyocytes in the zebrafish heart, and co‐ordinates with p53α to promote cell survival, thus contributing to myocardial regeneration (Ye *et al*, 2020). The mechanism of zebrafish cardiac regeneration also involves the MDM2‐mediated regulation of p53α (Shoffner *et al*, 2020). Together, the data suggest that heart regeneration might require fine‐tuning of the p53 isoform network. These mechanisms are likely to be conserved in mammalian cells as p53 has been shown to regulate the cardiac transcriptome in mice (Mak *et al*, 2017; Xiao *et al*, 2017).

p53 isoforms may also be involved in brain cell regeneration. Seminal studies indicated that p53 activity is increased in neuron precursors of the developing mouse brain, while p53 activity is reduced in neurons undergoing terminal differentiation (Rogel *et al*, 1985; Schmid *et al*, 1991). Moreover, several p53 isoforms are expressed in normal human foetal brain (Bourdon *et al*, 2005). Using mouse and zebrafish models, several studies have demonstrated that the regenerative capacity of neural precursor and axon regeneration is regulated by altering the p53 isoform ratio. During ageing, this regulatory mechanism deteriorates, resulting in disruptions in the ability of stem cells to proliferate leading to neurodegeneration (Medrano *et al*, 2009; Ungewitter & Scrable, 2010; Takahashi *et al*, 2013; Zhao *et al*, 2021). Interestingly, as in heart regeneration, the MDM2 pathway controls p53 network activity in axonal regeneration, sprouting and functional recovery after brain injury (Joshi *et al*, 2015).

Furthermore, highlighting the importance of the p53 isoform network, primary human astrocytes undergoing cellular senescence showed diminished expression of Δ133p53 and increased expression of p53β, while restoring expression of Δ133p53 in neurotoxic astrocytes induced neurotropic growth factors and repressed SASP, resulting in neuroprotection (Turnquist *et al*, 2016, 2019). In addition, overexpression of Δ133p53β in breast cancer cells also promotes a cancer stem cell phenotype by increasing the levels of differentiation gene products *SOX 2*, *OCT3/4* and *NANOG* (Arsic *et al*, 2015). Finally, mouse cells expressing p53Ψ are able to reprogramme cells to promote an invasive phenotype (Senturk *et al*, 2014). These studies show that p53 and isoforms contribute to several processes involved in tissue regeneration to maintain cellular and organismal homeostasis.

## p53, isoforms and the immune response

As well as regulating cellular homeostatic processes in response to a variety of stresses, there is considerable evidence that p53 and isoforms are central to organismal homeostasis by virtue of regulating the immune response ((Joruiz & Bourdon, 2016), Fig 1). Indeed, the data from the shrimp (Li *et al*, 2017) indicate that altering the pattern of isoform expression shifts the balance of the p53 network from regulating cellular homeostasis (controlling virus replication by LvFLp53) to organismal homeostasis (controlling inflammation by LvΔNp53). Similarly, a role for Δ133p53 in influencing the immune response comes from the observation that single‐nucleotide polymorphism (SNP) combinations in the *Δ133TP53* promoter/enhancer region (Marcel *et al*, 2010b) are linked with elevated *Δ133TP53* mRNA levels that are strongly associated with infiltration of immunosuppressive cells in several types of human cancers (Eiholzer *et al*, 2020). Prostate and brain cancers with elevated *Δ133TP53* mRNA levels have increased the numbers of immunosuppressive macrophages and CD4+ T cells (Kazantseva *et al*, 2018a, 2019) and have high levels of Programmed Cell Death 1 Ligand 1 (PD‐L1), encoding one of the surface molecules that inhibit anti‐tumour T‐cell responses (Karwacz *et al*, 2011). Moreover, Δ133p53β was found to directly increase PD‐L1 mRNA and protein in engineered cell lines (Kazantseva *et al*, 2019).

In addition, p53‐null mice show increased susceptibility to inflammation, auto‐immunity and cancer (Donehower *et al*, 1992; Okuda *et al*, 2003; Zheng *et al*, 2005; Guo *et al*, 2017) and various studies have shown that loss of p53 in myeloid cells can promote an immunosuppressive environment (Lowe & Ruley, 1993; Zheng *et al*, 2005; Guo *et al*, 2017). Other studies have shown that p53 limits T‐cell proliferation (Watanabe *et al*, 2014) and deletion of p53 in T cells results in an inflammatory phenotype and spontaneous autoimmunity (Zhang *et al*, 2011; Kawashima *et al*, 2013). Δ133p53 and p53β appear to be physiological regulators of proliferation and senescence in human T cells (Mondal *et al*, 2013), and when engineered to express Δ133p53α, there was reduced cell surface expression of PD‐1 and TIGIT (T‐cell immunoreceptor with Ig and ITIM domains) (Legscha *et al*, 2018). In a follow‐up study, this group also showed that the engineered T cells had a lower frequency of senescent‐like CD57+ and CD160+CD8+ T cells and an increased number of less differentiated CD28+ T cells (Legscha *et al*, 2021). These cells also had enhanced proliferative capacity, elevated cytokine secretion, similar to Δ122p53 mice (Slatter *et al*, 2011; Roth *et al*, 2016) and improved T‐cell killing. The above studies provide compelling evidence that p53 and its isoforms play important roles in modulating different aspects of the immune response, and indeed, they may be essential for sustaining a T‐cell response. Thus, the p53 network is a key component of immune system homeostasis (Fig 1).

## p53, isoforms and inflammatory signalling

### Nuclear factor kappa B signalling

Nuclear factor kappa B (NF‐κB) is a family of transcription factors that regulate a large number of genes involved in immune processes. Well‐recognized functions of NF‐κB are induction of pro‐inflammatory genes in innate immune cells, regulating T‐cell activation, differentiation and effector function and activation of inflammasomes (reviewed in Liu *et al*, 2017b). Thus, it is not surprising that perturbation of NF‐κB signalling results in chronic inflammatory disease (Pasparakis, 2009). It is well established that p53 inhibits inflammation by acting as an antagonist of NF‐κB (Komarova *et al*, 2005; Carrà *et al*, 2020), although co‐operation between p53α and NF‐κB has also been reported (Schneider *et al*, 2010; Liang *et al*, 2013; Iannetti *et al*, 2014; Lowe *et al*, 2014; Machado *et al*, 2018; Carrà *et al*, 2020), but this may vary depending on the cell type and stress stimulus. The cross talk between p53 and NF‐κB may also be modulated by a common SNP in the human *TP53* gene, resulting in either proline or arginine at position 72 of p53 (Luo *et al*, 2001). Transgenic mouse studies showed increased NF‐κB‐dependent inflammatory gene expression with the proline variant of p53 and enhanced response to lipopolysaccharide challenge (Luo *et al*, 2001). These residues overlap with a p53‐responsive enhancer/promoter in human *TP53* gene, which in combination with SNPs in the 3'UTR of the *Δ133TP53* transcript, are associated with increased *Δ133TP53* transcription (Mechanic *et al*, 2007; Bellini *et al*, 2010; Eiholzer *et al*, 2020). These data further suggest that p53 isoforms play an important role in the crosstalk between p53 and NF‐κB pathways. Powerful evidence for this comes from the studies conducted in *H. pylori*‐infected gastric cancer (Wei *et al*, 2010, 2012; Zhang *et al*, 2017). These studies showed that both NF‐κB and Δ133p53 are upregulated and play an important role in the development of gastritis and gastric cancer (Wei *et al*, 2010, 2012; Zhang *et al*, 2017). Moreover, they also demonstrated that the inhibition of NF‐κB p65 subunit resulted in down‐regulation of Δ133p53 expression and prevented proliferation, further reinforcing the cross‐talk between Δ133p53 and NF‐κB in gastritis‐associated cancer (Zhang *et al*, 2017). Another study found that Δ133p53 levels were elevated and p53β levels reduced in gastric adenocarcinomas compared to atrophic and superficial gastritis (Ji *et al*, 2015). The Δ122p53 mice also showed elevated levels of multiple serum cytokines including IL‐6 that are downstream targets of the NF‐κB and JAK/STAT3 pathways (Campbell *et al*, 2018). Moreover, the deletion of IL‐6 in these mice reduced the incidence of tumours and metastatic frequency. These data not only show that Δ133p53 increases NFκB signalling, but that sustained expression and the resulting inflammation have pathological consequences.

Serine and arginine‐rich splicing factor 1 (SRSF1) is an essential splicing factor and changes in the expression of this protein are associated with aberrant splicing in various diseases (Zheng *et al*, 2020). SRSF1 is known to play an important role in the maintenance of genomic stability, cell viability and cell‐cycle progression (Zheng *et al*, 2020). SRSF1 facilitates the production of type I IFNs recognized by the cytoplasmic pattern recognition receptor, RIG1, in psoriatic lesions (Xue *et al*, 2015). SRSF1‐mediated production of type I IFNs also prevents the development of systemic lupus erythematosus (SLE) by restraining T‐cell activation (Katsuyama *et al*, 2019) and is required for neuro‐immune suppression of the human neurotropic JC virus (JCV) (Sariyer *et al*, 2016). Human aortic smooth muscle cells (HASMCs) expressing SRSF1 showed high levels of Δ133p53α isoform and SRSF1‐deficient mice had lower levels of Δ157p53 (orthologue of Δ133p53) compared to controls. SRSF1 mediated upregulation of Δ133p53 promotes proliferation and migration observed during wound healing by inducing early growth response protein 1/Kruppel‐like factor 5 (EGR1/KLF5) pathway (Xie *et al*, 2017), resulting in induction of NF‐κB. In addition, SRSF1 (and SRSF3) inhibit(s) the alternative splicing of the exon 9β/γ of *TP53* gene preventing the induction of senescence (Tang *et al*, 2013; Marcel *et al*, 2014). Collectively these studies suggest that SRSF1 and SRSF3 are important in the mediation of cross‐talk between p53 and NF‐κB pathways by regulating the ratio of Δ133p53 to p53β.

### Interferon signalling

Interferons (IFNs) are cytokines expressed by cells as the first line of defence against viral infections during immune surveillance. IFN cytokines can be broadly classified into two classes, type I (IFNα, IFNβ, IFNε, IFNκ and IFNω) and type II IFNs (IFNγ) (Platanias, 2005). Both type I and type II IFNs signal via their respective receptors by interacting with a member of the JAK family and activation of the JAK/STAT pathway (Platanias, 2005). It has been shown that p53 induces the expression of type I IFN (Muñoz‐Fontela *et al*, 2016) and a number of IFN‐responsive genes (Rivas *et al*, 2010) as well as multiple Toll‐like receptor genes (Shatz *et al*, 2012) during IAV infection. Induction of type I IFN can promote p53α‐dependent apoptosis (Yuan *et al*, 2016; Dierckx *et al*, 2017) or senescence (Moiseeva *et al*, 2006; Kim *et al*, 2009). The regulation is complex, however, as it has also been reported that p53α can inhibit the IFN response by inhibiting STAT1, the transcription factor required to transactivate IFN inducible genes (Cheon *et al*, 2013).

A role for p53 isoforms in IFN signalling has been indicated by several studies. The RNA helicases DHX15 (McElderry *et al*, 2019) and DDX5 (Moore *et al*, 2010) are involved in activating an innate immune response to RNA virus infections, while inducing type I and II IFN (Moore *et al*, 2010; Wang *et al*, 2015; McElderry *et al*, 2019; Zan *et al*, 2020). Of interest, *Dhx15* ^−/−^ zebrafish embryos exhibited reduced expression of exons 1–4 of Zp53 and increased expression of Δ113p53 (McElderry *et al*, 2019). Moreover, in breast cancer cells, an inverse correlation was shown between p68 expression (encoded by DDX5) and Δ133p53 (Moore *et al*, 2010). Δ133p53α, p68 and p53α formed a complex and Δ133p53α inhibited the ability of p68 to stimulate p53‐dependent transcription (Moore *et al*, 2010; Zan *et al*, 2020). Thus, the Δ133p53 isoform appears to negatively impact DHX15 and DDX5 regulation of IFN signalling important in antiviral immunity. However, our own studies have shown that this isoform stimulates IFN signalling. Microarray analysis of splenocytes from Δ122p53 mice showed an enrichment for IFN pathways (Slatter *et al*, 2011, 2015; Campbell *et al*, 2012) and serum from the mice showed increased levels of pro‐inflammatory cytokines including IL‐6, TNF‐α and IFN‐γ and chemokines including GM‐CSF and CCL2 (Slatter *et al*, 2011; Campbell *et al*, 2012, 2018). Moreover, in a subset of breast cancers with mutant p53, a bioinformatic analysis showed that Δ133p53 transcripts were associated with an IFN‐γ signature and good patient prognosis (Mehta *et al*, 2018). Collectively, these results suggest that activation of IFN signalling may depend on the balance of p53 isoforms in different cell types.

### JAK/STAT and Rho/ROCK signalling

The JAK/STAT signalling pathway has also been implicated in inflammation, specifically in autoimmune disease (reviewed in Banerjee *et al,* 2017). Evidence that p53 isoforms play an important role in JAK/STAT signalling comes from the Δ122p53 mouse model of Δ133p53 (Slatter *et al*, 2011; Roth *et al*, 2016; Campbell *et al*, 2018). The Δ122p53 mice developed tumours along with widespread inflammatory conditions such as lymphoid aggregates in several tissues and vasculitis. Furthermore, the serum from Δ122p53 mice showed elevated levels of multiple pro‐inflammatory cytokines and chemokines as did the culture media from Δ122p53‐expressing MEFs (Slatter *et al*, 2011; Roth *et al*, 2016). MEFs expressing Δ122p53 and osteosarcoma Saos‐2 cells expressing Δ133p53 promoted invasion and metastasis which was prevented with inhibitors of both JAK/STAT and Rho/ROCK pathways (Campbell *et al*, 2018). The importance of this inflammatory signalling was further demonstrated when the mice were crossed on to an IL‐6 null background. Δ122p53 mice that lacked IL‐6 showed reduced activation of the JAK/STAT and Rho/ROCK signalling pathway and had a reduced incidence of tumours and metastases (Campbell *et al*, 2018). Thus, a chronic imbalance of p53 isoform may lead to malignant disease driven by inflammatory mediators. Of interest, Ewing Sarcoma cells that have elevated levels of Δ133p53 were shown to induce hepatocyte growth factor (HGF) secretion, resulting in tumour growth and metastasis (Charan *et al*, 2020) and cooperation between HGF and IL‐6 resulted in proliferation and migration of myeloma cells (Hov *et al*, 2009). IL‐6 is elevated in multiple cancers, is known to be involved in invasion and metastasis (Jayatilaka *et al*, 2017), promotes HGF production (Coudriet *et al*, 2010) and signals via the activation of the JAK/STAT, PI3K, MAPK and AMPK pathways in a cell type‐dependent manner. Thus, Δ133p53 appears to increase the expression of signalling molecules, such as cytokines, that promote inflammation that in turn drives cancer progression via activation of JAK/STAT and Rho/ROCK signalling pathways.

### Tumour necrosis factor signalling

Another immune signalling pathway affected by p53 and isoforms is that controlled by tumour necrosis factor (TNF). TNF is a monocyte‐derived cytokine that stimulates the immune system to mount an acute phase reaction, which has the ability to destroy tumour vasculature, induce haemorrhagic necrosis and synergize with various chemotherapeutic reagents (Balkwill, 2009). TNFα/IFNγ synergistically activates c‐Jun N‐terminal kinase/stress‐activated protein kinase (JNK/SAPK) to promote apoptosis of pancreatic β‐cells via activation of the p53 pathway together with ROS (Kim *et al*, 2005). Studies using gastric cancer cells treated with recombinant human TNF either alone or in combination with 5‐flurouracil resulted in reduction of Δ133p53 levels and an induction of p53α resulting in apoptosis (Shang *et al*, 2015), suggesting Δ133p53 may function to inhibit/moderate p53α‐promoted, TNF‐induced apoptosis.

TNFα is a potent inhibitor of angiogenesis both *in vitro* and *in vivo*. Angiogenesis is essentially stimulated by the presence of hypoxic regions within a tissue and angiogenesis and inflammation are tightly linked though the functions of TNFα (Fiedler *et al*, 2006; Imhof & Aurrand‐Lions, 2006). Which process is favoured appears to be dependent on TNFα concentration. p53 is known to be involved in inhibition of angiogenesis by regulation of hypoxia, inhibiting the production of pro‐angiogenic factors (e.g. VEGFA) and by increasing the production of anti‐angiogenic factors (e.g. MMP2) (Teodoro *et al*, 2007). On the other hand, Δ133p53α and Δ133p53β both increase the levels of several angiogenic factors including VEGFA (Kazantseva *et al*, 2019) and Δ133p53 directly induces angiogenesis *in vivo* and activates distinct angiogenic signalling pathways (Bernard *et al*, 2013). In addition, abnormal angiogenesis was observed in many organs of the Δ122p53 mice in areas with inflammatory lesions (Slatter *et al*, 2011). Collectively, these data show that abnormal and sustained expression of Δ133p53 isoforms alter normal angiogenic homeostasis, which very likely promote cancer progression.

## Summary

**Figure 4:**
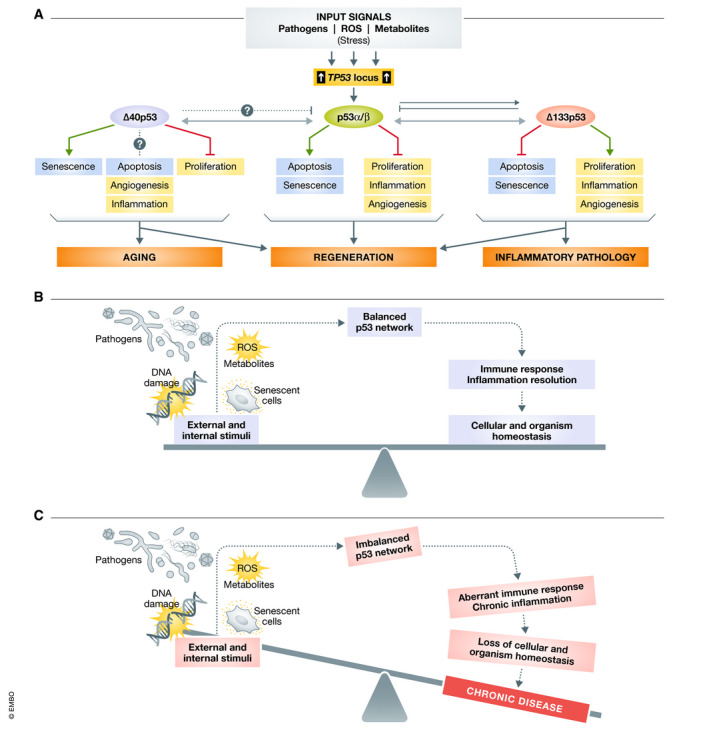
Model illustrating the role of the p53 network in maintaining homeostasis (A) Schematic showing the role of different p53 isoforms in biological processes and their influence on each other. (B) Cells and organisms are continuously exposed to stimulus from external and internal sources. Under physiological conditions, a balanced p53 network responds to these stimuli and regulates immune response and inflammation to maintain cellular and organismal homeostasis. (C) Prolonged exposure to a variety of external and internal stimuli causes an imbalance in the p53 network, which in turn results in aberrant immune response and chronic inflammation. These changes result in loss of cellular and organismal homeostasis resulting in pathologies associated with chronic diseases.

The p53 isoform field is arguably the least well‐understood area of p53 biology (see also Box 1). Whilst there are many published studies implicating one or more p53 isoforms in multiple biological processes, they vary considerably in detail and there is often a paucity of mechanistic information, including how the isoforms are turned on or activated. One feature all the isoforms (except p53Ψ) have in common is that they function as transcription factors. p53β functions independently of p53α and has very similar biological activities. Δ40p53 can alter p53α to induce different gene sets from p53α alone, but can also affect gene transcription independently of p53α. Similarly, whilst the Δ133p53 family can modulate p53α, it has p53α‐independent transactivation capacity with a very different transcriptional target repertoire. There is also evidence that the isoform families can modulate each other. Given this conserved ability of p53 isoforms to function as transcription factors and the many p53REs scattered throughout the human genome, present in endogenous retroviruses and other TEs, suggests that the p53 network can shape the transcriptional programme of cells. This provides an explanation for how the p53 network can contribute to multiple adaptive functions that have an impact on homeostasis in response to many input signals. An outline of such contributions where the data are available is shown in Fig 4A.

Box 1 **In need of answers**

1. How is the *TP53* isoforms network regulated? What signals initiate transcription? Are there signals that activate the entire network and others that are isoform specific? Are viruses/pathogens the principal signals initiating isoform transcription and regulating function? Is FLp53 important?
2. What initiates *TP53* splicing? How is this regulated?
3. How is the *TP53* isoform network coordinated to regulate biological outcomes? How important are post‐translational modifications in modulating isoform functions? How important are isoform interactions? *(Some of these questions could perhaps be addressed by treating cells with different stresses accompanied by long‐range RNA‐sequencing and sensitive mass spectrometry)*.
4. How do the isoforms regulate downstream genes/proteins? What co‐factors are required? *(These questions may be addressed using ATAC‐sequencing)*.
5. What are the key downstream genes targeted by one or more isoforms, which are presumably cell specific? *(This question could be addressed using CRISPR/Cas9 gene knock out screens in different cells)*.
6. How do isoform functions change (i.e. the transcriptional profile) when they are encoded off a mutated *TP53* gene (as is the case in cancers and in some inflammatory disorders)? *(This could be done using RNA‐sequencing, comparing cells with different TP53 mutations)*.
7. How does the *TP53* network contribute to tissue homeostasis in response to cellular stresses? What is the role of transposable elements? *(Perhaps some answers to this could be obtained as under question 3 using long‐range RNA sequencing)*.
8. How important are the isoforms in development, tissue regeneration and immune modulation? What are the underlying mechanisms? *(These questions would need to be addressed using transgenic animals in which individual isoforms are mutated or deleted using targeted mutagenesis)*.

Having a single gene network regulating homeostasis ensures coordination of responses and adaptation to changing environmental conditions enabling the survival of the individual and its offspring. This is well illustrated in how the p53 network responds to infection. Different components of the network simultaneously aid and moderate pathogen replication; alter cell physiology to cope with pathogen load; alter cell lifespan to allow pathogen replication; and trigger inflammation to limit pathogen spread. In this way, stress responses at the cellular level are linked to those at the level of the whole organism ensuring an overall homeostatic balance. As well as infection, the p53 network responds to, and influences, many fundamental responses of cells to changing environmental conditions (e.g. nutrient deprivation; changes in pH, temperature, oxygen levels, osmolarity and radiation exposure) by inducing survival, repair, proliferation, senescence, differentiation or cell death programmes and by modulating immune cell function and surveillance affecting the biology of the entire organism (Fig 1). As the p53 network is responsive to many different environmental alterations, adapting cell and tissue functions accordingly, it is necessarily very fine‐tuned. Thus, it is inevitable that extended periods of imbalance in the network lead to pathology (Fig 4B and C). This is notable for the Δ133p53 isoform family which, when chronically over‐expressed, promotes ageing‐related conditions such as senescence, other physiological anomalies of cell migration, unchecked cell proliferation and angiogenesis, long‐term inflammatory conditions and cancer. As well, chronic over‐expression of Δ40p53 also results in pathology associated with reduced proliferation, senescence and ageing. Thus, the critical adaptive functions of the p53 network at the cell and organism level provide an explanation for its extraordinary conservation from relatively simple multicellular to very complex organisms.

## Conflict of interest

The authors declare that they have no conflict of interest.
